# Dead End Metabolites - Defining the Known Unknowns of the *E*. *coli* Metabolic Network 

**DOI:** 10.1371/journal.pone.0075210

**Published:** 2013-09-23

**Authors:** Amanda Mackie, Ingrid M. Keseler, Laura Nolan, Peter D. Karp, Ian T. Paulsen

**Affiliations:** 1 Department of Chemistry and Biomolecular Sciences, Macquarie University, Sydney, New South Wales, Australia; 2 SRI International, Menlo Park, California, United States of America; Hospital for Sick Children, Canada

## Abstract

The EcoCyc database is an online scientific database which provides an integrated view of the metabolic and regulatory network of the bacterium *Escherichia coli* K-12 and facilitates computational exploration of this important model organism. We have analysed the occurrence of dead end metabolites within the database – these are metabolites which lack the requisite reactions (either metabolic or transport) that would account for their production or consumption within the metabolic network. 127 dead end metabolites were identified from the 995 compounds that are contained within the EcoCyc metabolic network. Their presence reflects either a deficit in our representation of the network or in our knowledge of *E. coli* metabolism. Extensive literature searches resulted in the addition of 38 transport reactions and 3 metabolic reactions to the database and led to an improved representation of the pathway for Vitamin B12 salvage. 39 dead end metabolites were identified as components of reactions that are not physiologically relevant to *E. coli* K-12 – these reactions are properties of purified enzymes *in vitro* that would not be expected to occur *in vivo*. Our analysis led to improvements in the software that underpins the database and to the program that finds dead end metabolites within EcoCyc. The remaining dead end metabolites in the EcoCyc database likely represent deficiencies in our knowledge of *E. coli* metabolism.

## Introduction

Symbolic systems biology is the application of logic-based computational methods to the systems-level analysis of an organism. Previously, several types of symbolic systems biology approaches have provided novel biological insights. For example, metabolic pathway analysis of genomes can be used to identify reactions within metabolic pathways that have no associated enzyme (“pathway holes”) [1], thus motivating a search for gene(s) within the organism that code for the missing enzyme. Conversely, orphan enzymes are enzymes whose biochemical function has been demonstrated experimentally, but for which the associated gene has not been identified [2]. In both cases, the explicit identification of holes in our knowledge spurs a whole series of new investigations.

**Figure 1:**
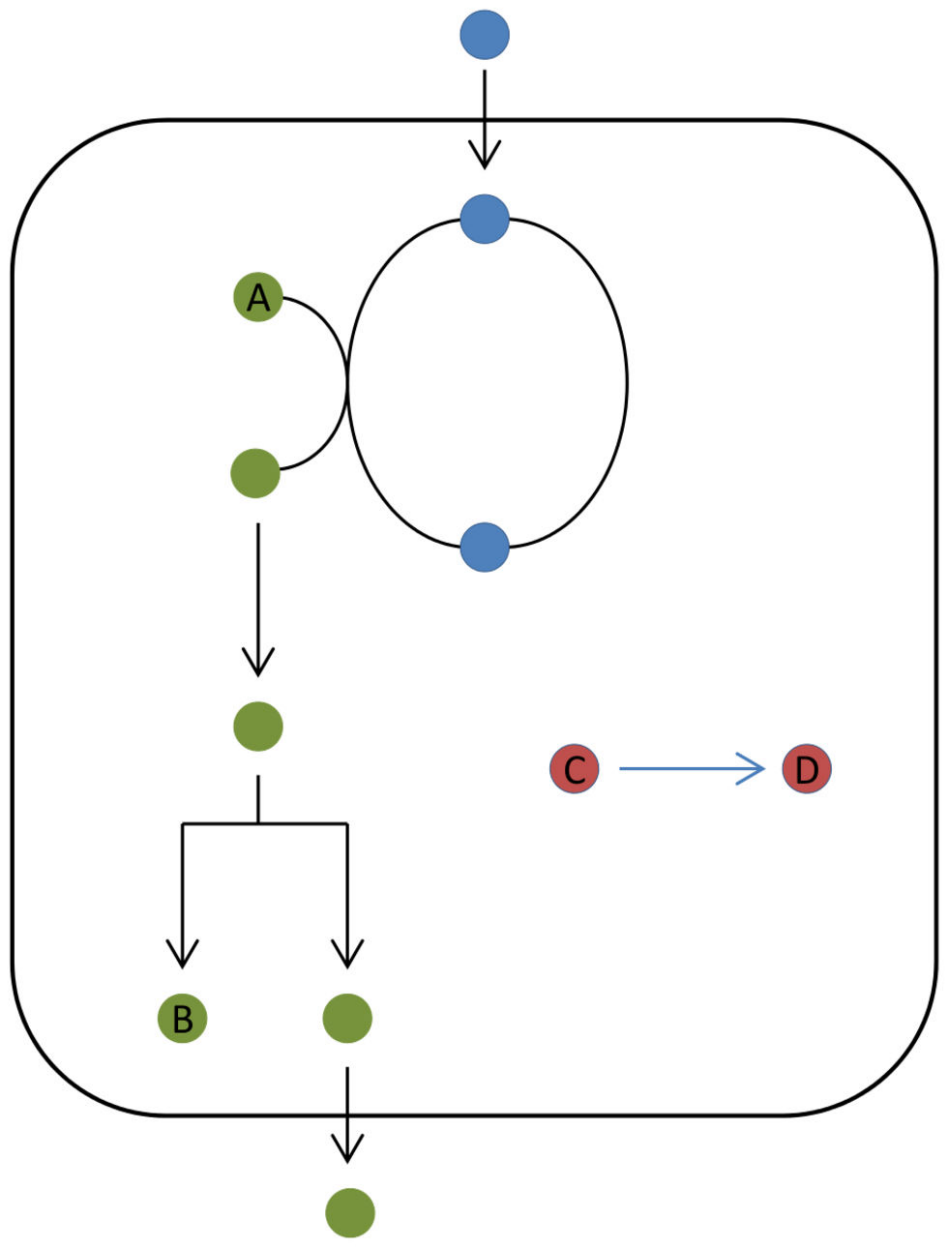
Representation of generic dead end metabolites (A, B, C and D) within a metabolic network. The compounds labeled A and C are neither produced nor transported by any other reaction within the network while the compounds B and D are neither consumed nor transported by any other reaction within the network.

A dead-end metabolite (DEM) is defined as a metabolite that is produced by the known metabolic reactions of an organism and has no reactions consuming it, or that is consumed by the metabolic reactions of an organism and has no known reactions producing it, and in both cases has no identified transporter (Figure 1). DEMs are thus isolated compounds within a metabolic network. In some cases, DEMs reflect a deficit or an error in how a metabolic database represents knowledge from the scientific literature, and alerts us to the need for further curation of the database. In other cases this systems-level analysis alerts us to areas where more experimental research is required. In the latter case DEMs act as signposts to the ‘known unknowns’ of metabolism.

Our DEM analysis of *Escherichia coli* K-12 MG1655 was conducted using EcoCyc (http://EcoCyc.org), an online encyclopedia of *E. coli* K-12 biology that provides an integrated view of the genome, genes and gene products, and the metabolic and regulatory networks of this important model organism [3]. EcoCyc combines computable representations of these biological features of *E. coli* K-12, along with detailed summaries from manual literature curation. In release version 17.0 (March, 2013), EcoCyc contained 1497 metabolic enzymes and 268 transporters catalysing a total of 2175 reactions. The database contains 2392 compounds of which 995 are directly involved in reactions (the remainder being, for example, enzyme cofactors or inhibitors). EcoCyc version 17.0 also cites 24,391 publications from the literature. In addition to being a comprehensive reference resource, EcoCyc also provides tools that can be used for computational exploration within the database including multiple search tools and the identification of DEMs [4] (see EcoCyc website command Tools → Dead-end metabolites).

This project was undertaken to identify and analyse the dead end metabolites within the EcoCyc database. Our analysis led to the improved curation of many compounds within the database and also to improvements within the Pathway Tools software that underpins the database. We were able to resolve the dead end status of a large number of compounds through the addition of previously missing metabolic or transport reactions. As a result we are able to more accurately define the true DEMs within the EcoCyc database and by extension the ‘known unknowns’ within the metabolic machinery of the model organism *E. coli* K-12.

## Results

### Identification of DEMs in EcoCyc

DEMs within the EcoCyc database were identified using the DEM finder tool. In EcoCyc, metabolites may be reactants or products of reactions that occur within metabolic pathways defined within the database, or metabolites may form part of isolated reactions that are not contained within defined pathways. The DEM finder tool in EcoCyc can be customised to identify only those compounds that exist within metabolic pathways (pathway DEMs) or to include DEMs that come from reactions occurring outside pathways (non-pathway DEMs). Participation in metabolic pathways may make pathway DEMs rare, but also more likely to be physiologically relevant. A search of 271 metabolic pathways yielded 32 DEMs while a search of 393 isolated reactions within EcoCyc returned a further 123 compounds. 28 of these compounds were lacking proper classification within the EcoCyc database and could be resolved by correcting this omission. For example, correct classification of the compound “methylphosphonate” as a child of the class “alkylphosphonates” meant that it was recognised by the EcoCyc software as a substrate of the phosphonate ABC transporter, thus resolving its dead end status. The remaining 127 compounds (Table 1) were the subject of further analysis.

**Table 1:** Dead end metabolites from the EcoCyc database.

(2R,4S)-2-methyl-2,3,3,4-tetrahydroxytetrahydrofuran (AI-2)^*p*^	allantoin ^*p*^	maltopentaose
(E)-3-(methoxycarbonyl)pent-2-enedioate	aminoacetaldehyde ^*p*^	methanol ^*p*^
(R)-beta-lysine	aminomethylphosphonate	methyl red
(R)-malate	an unknown C3 fragment	methyl-1,4-benzoquinol
(R)-pantolactone	benzaldehyde	methyl-1,4-benzoquinone
1-chloro-2,4-dinitrobenzene (CDNB)	CDP-choline	N,N'-dimethyl-p-phenylenediamine
1-deoxy-D-xylulose	cholate	N-alpha-acetyl lysine methyl ester
2,3-diaminopropionate	cis-vaccenate ^*p*^	N-ethylmaleimide
2,4-dinitrophenyl-S-glutathione	CO	N-ethylsuccinimide
2-aminobutyrate	cobinamide ^*p*^	nicotinamide riboside
2-aminomalonate-semialdehyde	cofactor	nigerose
2-dehydropantolactone	corresponding carbamoyl amino acid	N-methyltryptophan
2-deoxy-D-glucose	Cr3+	NMNH
2-deoxy-D-glucose 6-phosphate	Cr6+	octanoate ^*p*^
2-deoxygluconate	curcumin ^*p*^	oxalate
2-protocatechuoylphloroglucinolcarboxylate	D-4-hydroxy-2-keto-glutarate (KHG)	oxamate ^*p*^
3,4-dihydroxyphenylacetate	d-biotin d-sulfoxide	phenylethylamine ^*p*^
3,4-dihydroxyphenylacetyl-CoA	D-galactono-1,4-lactone	phenylhydantoin
3,5-tetradecadienoate^*p*^	diacetyl	pre-cofactor
3-alpha,12-alpha-dihydroxy-7-oxo-5-beta-cholanate	diacylglycerol pyrophosphate	pseudouridine ^*p*^
3-chloro-D-alanine	dihydrolipoamide	psicoselysine ^*p*^
3-dehydro-2-deoxy-D-gluconate	ethanolamine ^*p*^	pyrazinamide
3-hydroxy-5-cis-tetradecenoyl-CoA^*p*^	ethyl-(2R)-methyl-(3S)-hydroxybutanoate	pyrazinoate
3-hydroxypropionate^*p*^	ethyl-2-methylacetoacetate	queuine
3-hydroxy-trans-cinnamate^*p*^	fructoselysine ^*p*^	quinate
3-mercaptopyruvate	GDP-alpha-D-glucose	S2- ^*p*^
3-methylcrotonyl-CoA	glycerol 2-phosphate	S-adenosyl-4-methylthio-2-oxobutanoate ^*p*^
3-phenylpropanoate^*p*^	GMP-lysine	salicyl alcohol
3-sulfinoalanine (AKA cysteine sulfinic acid)	heptosyl-KDO2-lipid IVA	S-carboxymethyl-D-cysteine
4-(2-aminophenyl)-2,4-dioxobutanoate	hydroxylamine	selenate1,3-propanediol
4-coumarate	hydroxymethylpyrimidine (HMP)^*p*^	selenite
4-coumaroyl-CoA	hydroxypropionaldehyde	S-methyl-5-thio-D-ribose ^*p*^
4-methyl-5-(beta-hydroxyethyl)thiazole (THZ)^*p*^	isovaleryl-CoA	S-methyl-L-methionine
4-nitrobenzaldehyde	kynurenine	tetrahydrocurcumin ^*p*^
4-nitrobenzyl alcohol	L-glyceraldehyde 3-phosphate	tetrahydromonapterin ^*p*^
5', 5'-diadenosine triphosphate	L-idarate	thioglycolate
5,6,7,8-tetrahydropteridine	linear dimeric GMP	trans-aconitate
5,6-dimethylbenzimidazole (DMB)^*p*^	lipoamide	trans-cinnamate ^*p*^
6,7-dihydropteridine	lipoate ^*p*^	UDP-[N-acetyl-D-glucosamine]n
acetoacetate ^*p*^	L-methionine sulfoxide	urate ^*p*^
acetylmaltose	L-rhamnonate	urea ^*p*^
adenosine thiamine triphosphate	L-selenocysteine	
adenosylcobalamin-5'-phosphate	L-threo-3-phenylserine	

^*p*^ DEMs derived from within EcoCyc metabolic pathways.

**Figure 2:**
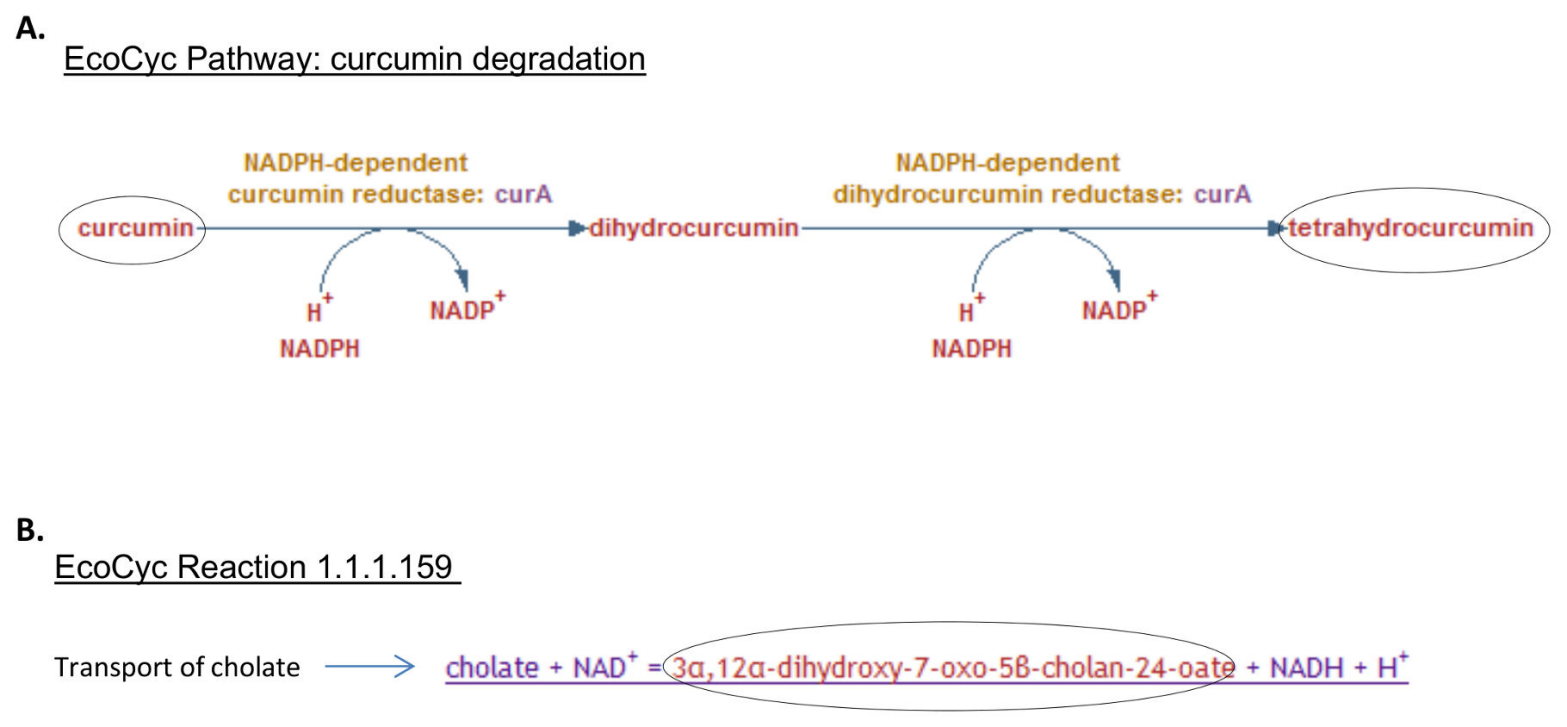
Dead end metabolites in EcoCyc. **A**. The EcoCyc curcumin degradation pathway in EcoCyc produces two dead end metabolites (circled). **B**. An isolated EcoCyc reaction (catalysed by *E. coli* 7-α-hydroxysteroid dehydrogenase) produces the single dead end metabolite, 3α,12α-dihydroxy-7-oxo-5β-cholan-24-oate.

Examples of DEMs and the EcoCyc reactions from which they derive are shown in Figure 2. Both curcumin (a secondary metabolite produced in turmeric and other plant species, see http://www.metacyc.org/META/NEW-IMAGE?type=PATHWAY&object=PWY-6432) and tetrahydrocurcumin are considered to be pathway DEMs in EcoCyc because the database contains no other reactions for these molecules – it does not describe the production nor transport of curcumin, nor the fate of tetrahydrocurcumin. In contrast, the compound 3α,12α-dihydroxy-7-oxo-5β-cholan-24-oate (product of an *E. coli* 7-α-hydroxysteroid dehydrogenase, HdhA) is a DEM from an isolated reaction in EcoCyc. HdhA acts on cholate, which is thought to cross the inner membrane of *E. coli* [5]; the fate of 3α,12α-dihydroxy-7-oxo-5β-cholan-24-oate in the *E. coli* cell is unknown although it is known to be further metabolised by other anaerobic and facultative anaerobic bacteria in the human intestine (see http://metacyc.org/META/NEW-IMAGE?type=PATHWAY&object=PWY-6518).

### DEM analysis and resolution

The 127 DEMs identified in the EcoCyc database encompass a wide variety of compounds. As a necessary first step to understand the biochemistry of each compound and its metabolic context, the reactions containing the DEMs were identified within EcoCyc and any associated literature was reviewed. Extensive literature searches were then conducted to see if further information relating to the compound could be uncovered.

#### Resolution of DEMs through the addition of transport reactions

38 DEMs were resolved through the addition of transport reactions to the database (Table 2). 29 new transport reactions were created to represent the import of metabolites across the inner membrane (Table S1) and 9 reactions were created to represent export of metabolites from the cytoplasm (Table S2). Transport reactions were only added to EcoCyc in cases where we found substantiating evidence in the experimental literature (Table 2). Several of the references describing transport date back more than 30 years. For example, the experimental work reporting the production and excretion of urea into the culture media dates back to studies of putrescine biosynthesis in *E. coli* K-12 carried out in 1967 [6]. Similarly the ability of *E. coli* K-12 to use acetoacetate as a sole carbon source was reported by Pauli and Overath in 1972 [7]. For 15 of the 38 reactions, a specific transport protein had been characterised and reported in the literature, while in a further 4 cases the identity of membrane proteins responsible for transport had been predicted. For the remaining metabolites, no information on the mechanism of transport was located in the literature during this study, suggesting that these transport systems remain to be characterised. In these latter cases a transport reaction was added to the EcoCyc database but it was not associated with a specific transport protein.

**Table 2:** DEMs resolved by the addition of transport reactions to the EcoCyc database.

**Metabolite**	**Transport system**	**Literature reference**
acetoacetate	predicted short chain fatty acid transporter AtoE	[7]
phenylacetaldehyde	**-**	[25]
psicoselysine	psicoselysine transporter FrlA	[32]
fructoselysine	fructoselysine transporter FrlA	[33]
octanoate	**-**	[34]
3-phenylpropanoate	predicted 3-phenylpropanoate transporter HcaT	[35]
lipoate	**-**	[36]
cobinamide	vitamin B12 transport system	[37,38]
3-hydroxy-trans-cinnamate	3-hydroxycinnamate:H^+^ symporter MhpT	[39]
trans-cinnamate	**-**	[35,39]
(2R,4S)-2-methyl-2,3,3,4-tetrahydroxytetrahydrofuran (AI-2)	autoinducer-2 ABC transporter LsrACDB	[40]
4-methyl-5-(β-hydroxyethyl)thiazole (THZ)	**-**	[41]
5,6-dimethylbenzimidazole (DMB)	**-**	[8]
hydroxymethylpyrimidine (HMP)	**-**	[42]
pseudouridine	predicted pseudouridine transporter PsuT	[43]
ethanolamine	**-**	[44,45]
allantoin	predicted transporter YbbW	[46]
R-malate	C4 dicarboxylate/orotate transporter DctA	[47]
1-deoxy-D-xylulose	**-**	[48]
*d-*biotin-*d-*sulfoxide	**-**	[27,49]
glycerol-2-phosphate	glycerol-3-phosphate/glycerol-2-phosphate ABC transporter	[50]
nicotinamide riboside	nicotinamide riboside ABC transporter	[51]
S-methyl-L-methionine	S-methyl-L-methionine transporter	[52]
selenite	sulfate/thiosulfate/selenite/selenate ABC transporter	[53]
selenate	sulfate/thiosulfate/selenite/selenate ABC transporter	[53]
L-selenocysteine	**-**	[54]
cholate	**-**	[5]
L-glyceraldehyde 3-phosphate	glycerol 3-phosphate transport systems	[55,56]
aminomethylphosphonate	phosphonate ABC transporter	[57,58]
3,5-tetradecadienoate	**-**	[10,59]
3-hydroxypropionate	**-**	[60]
S-methyl-5-thio-D-ribose	**-**	[61]
urea	glycerol channel GlpF; passive diffusion	[6]
methanol	**-**	[62]
(2R,4S)-2-methyl-2,3,3,4-tetrahydroxytetrahydrofuran (AI-2)	quorum signal AI-2 exporter TqsA	[63]
acetylmaltose	**-**	[64]
salicyl alcohol	**-**	[65]
hydroquinone	**-**	[65]

#### Resolution of DEMs through the addition of metabolic reactions

The number of metabolic reactions added to EcoCyc as a result of DEM analysis was small. One case worth mentioning involved the DEM adenosylcobinamide-5-phosphate, whose presence alerted us to inaccuracies in our representation of the pathway of adenosylcobalamin (coenzyme B12) salvage in EcoCyc. Experimental work on this pathway is largely carried out in *Salmonella* *typhimurium* , which can synthesize adenosylcobalamin *de novo*, unlike *E. coli* K-12, which can synthesize cobalamin only when supplied with the intermediate compound cobinamide [8]. Despite this, the enzymes of the salvage pathway in *E. coli* are homologous to those from *S* *. typhimurium* and our DEM analysis revealed that the final steps of the pathway in *S* *. typhimurium* had recently been experimentally characterised [9]. Based on this we were able to correct our representation of the final two steps in the pathway of adenosylcobalamin salvage in EcoCyc (see http://ecocyc.org/ECOLI/NEW-IMAGE?type=PATHWAY&object=COBALSYN-PWY) and in the process resolve the dead end status of adenosylcobinamide-5-phosphate.

Another DEM, 3-hydroxy-5-cis-tetradecenoyl-CoA (product of a reaction catalysed by the FadB subunit of the fatty acid oxidation complex in *E. coli* K-12), revealed a deficiency in our link between two related pathways, oleate β-oxidation and fatty acid β-oxidation. This was remedied by the addition of the following two metabolic reactions [10]:

i) 3-hydroxy-5-cis-tetradecenoyl-CoA + NAD → 3-keto-5-cis-tetradecenoyl-CoA + NADH + H+ (an instance reaction of EC 1.1.1.211, catalysed by E. coli K-12 FadB)
ii) 3-keto-5-cis-tetradecenoyl-CoA + coenzyme A → 3-cis-dodecenoyl-CoA + acetyl-CoA (an instance reaction of EC 2.3.1.16, catalysed by E. coli K-12 FadA)

The product of EC 2.3.1.16, 3-cis-dodecenoyl-CoA, feeds into fatty acid oxidation (after conversion to a trans isomer) and thus the two reactions provide the direct link between oleate β-oxidation and fatty acid oxidation that had previously been lacking in the EcoCyc database.

One spontaneous reaction (trans-aconitate ↔ *cis*-aconitate; EC 5.3.3.7), representing formation of the DEM trans-aconitate from the citric acid cycle intermediate *cis*-aconitate [11], was also added to EcoCyc as a result of our DEM analysis.

#### DEMs that are not physiologically relevant

A large number of DEMs were associated with reactions that are not part of the normal physiology of *E. coli* K-12 or are of uncertain physiological relevance (Table 3). These reactions are properties of the purified enzyme *in vitro* that probably do not occur *in vivo*. Some of these reactions represent standard laboratory assays used to test for enzyme activity. For example, the compound 1-chloro-2,4-dinitrobenzene (CDNB) is used to assay glutathione S-transferase activity (EC 2.5.1.18) *in vitro* but CDNB is not membrane permeable [12] and thus is highly unlikely to be present within an *E. coli* cell. In many cases an enzyme catalysing a reaction containing non-physiological DEMS is also associated with a reaction that has physiological relevance to *E. coli*. For example, D-cysteine desulfhydrase (encoded by *dcyD*) is a cysteine-specific carbon-sulfur lyase with a role in the utilization of D-cysteine as a source of sulfur and is associated in EcoCyc with the physiologically relevant reaction EC4.4.1.15. However, the purified enzyme is also an effective 3-chloro-D-alanine dehydrochlorinase (EC 4.5.1.2) *in vitro* although it does not contribute to detoxification *in vivo* [13]. Association of DcyD with EC 4.5.1.2 creates two non-pathway DEMs in EcoCyc. In other cases, the non-pathway DEMs and their specific reactions are associated with enzymes whose true physiological role is unknown. *E. coli* proteins encoded by *ygeX*, *solA* and *hyuA* are examples of enzymes with associated reactions in EcoCyc, whose physiological role has not been characterised. One enzyme (AzoR) which had been characterised as an azoreductase *in vitro* was reannotated after a literature search revealed its physiologically relevant role to be an NADH: quinone reductase with a role in the protection against thiol stress [14].

**Table 3:** DEMs that are non-physiological or of uncertain physiological relevance in EcoCyc.

**Metabolites that are non-physiological**	**EcoCyc reaction and associated enzyme (if known**)
2-deoxy-D-glucose 6-phosphate; 2-deoxy-D-glucose	2-deoxy-D-glucose 6-phosphate + H_2_O ↔ phosphate + 2-deoxy-D-glucose(YniC)
(R)-pantolactone; 2-dehydropantolactone	(*R*)-pantolactone + NADP^+^ ↔ 2-dehydropantolactone + NADPH + H^+^
1-chloro-2,4-dinitrobenzene (CDNB); 2,4-dinitrophenyl-S-glutathione	1-chloro-2,4-dinitrobenzene + glutathione ↔ 2,4-dinitrophenyl-S-glutathione + chloride + H^+^ (YfcF, GstA)
3-chloro-D-alanine; S-carboxymethyl-D-cysteine; thioglycolate	3-chloro-D-alanine + thioglycolate ↔ S-carboxymethyl-D-cysteine + chloride + H^+^ (DcyD)
hydroxypropionaldehyde; 1,3-propanediol	3-hydroxypropionaldehyde + NADPH + H^+^ = 1,3-propanediol + NADP^+^(YqhD)
2,3-diaminopropionate	2,3-diaminopropanoate + H_2_O <=> 2 ammonia + pyruvate + H^+^ (YgeX)
Cr3+; Cr6+	2 NAD(P)H + Cr^6+^ + oxygen → 2NAD(P)^+^ + Cr^3^ + hydrogen peroxide (YieF)
methyl red; N,N'-dimethyl-p-phenylenediamine	methyl red + 2NADH + 2H^+^ = anthranilate + *N,N'*-dimethyl-*p*-phenylenediamine + 2NAD^+^ (AzoR)
N-methyltryptophan	*N*-methyltryptophan + oxygen + H_2_O → L-tryptophan + hydrogen peroxide + formaldehyde (SolA)
phenylhydantoin	phenylhydantoin + H_2_O ↔ corresponding carbamoyl amino acid (HyuA)
pyrazinamide; pyrazinoate	pyrazinamide + H_2_O = pyrazinoate + ammonia + H^+^ (PncA)
**Metabolites of uncertain physiological significance**	**EcoCyc reaction and associated enzyme (if known**)
3,4-dihydroxyphenylacetate; 3,4-dihydroxyphenylacetyl-CoA	3,4-dihydroxyphenylacetyl-CoA + H_2_O <=> 3,4-dihydroxyphenylacetate + coenzyme A + H^+^ (PaaI)
quercetin; 2-protocatechuoylphloroglucinolcarboxylate	quercetin + oxygen → 2-protocatechuoylphloroglucinolcarboxylate + carbon monoxide (YhhW)
4-(2-aminophenyl)-2,4-dioxobutanoate; kynurenine	2-oxoglutarate + kynurenine = L-glutamate + 4-(2-aminophenyl)-2,4-dioxobutanoate(AspC)
4-nitrobenzaldehyde; 4-nitrobenzyl alcohol	4-nitrobenzaldehyde + NADPH + H^+^ = 4-nitrobenzyl alcohol + NADP^+^(DkgB)
benzaldehyde; L-*threo*-3-phenylserine	L-threo-3-phenylserine = benzaldehyde + glycine(LtaE)
GMP-lysine; N-alpha-acetyl lysine methyl ester	GMP-N-ε-(N-α-acetyl lysine methyl ester) 5'-phosphoramidate + H_2_O → GMP + N- α-acetyl lysine methyl ester (HinT)
N-ethylmaleimide; N-ethylsuccinimide	*N*-ethylmaleimide + 2H^+^ = N-ethylsuccinimide (NemA)
methyl-1,4-benzoquinol; methyl-1,4-benzoquinone	methyl-1,4-benzoquinone + NADPH + 3H^+^ = methyl-1,4-benzoquinol + NADP^+^(YtfG)
nigerose	nigerose + H_2_O = 2α-D-glucose(YgjK)
5,6,7,8-tetrahydropteridine; 6,7-dihydropteridine	NAD(P)^+^ + 5,6,7,8-tetrahydropteridine ↔ NAD(P)H + 6,7-dihydropteridine + H^+^ (NfsB, Hmp)

**Figure 3:**
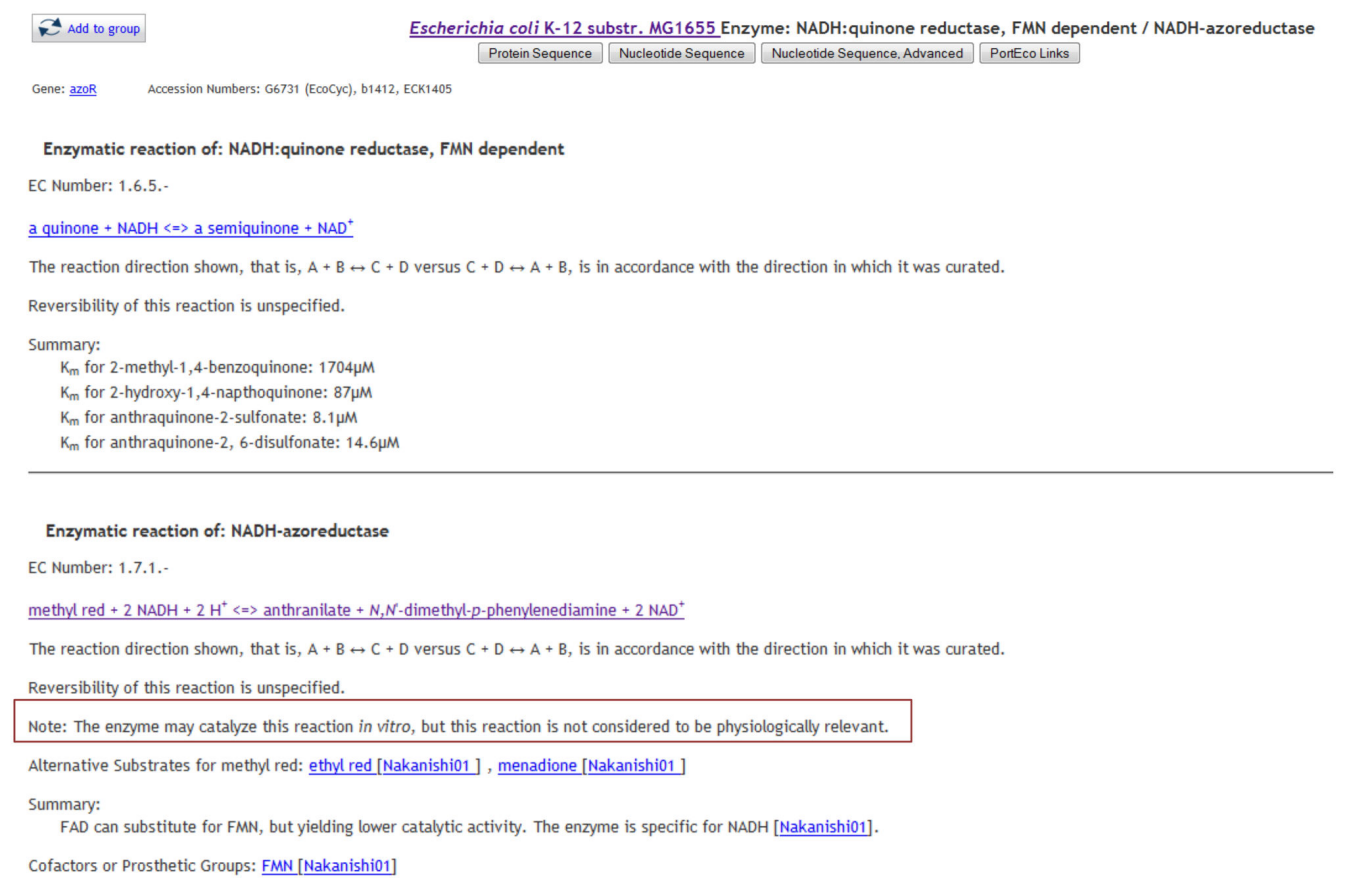
Reactions that are not part of the normal physiology of *E. coli* K-12 MG1655 can now be identified as such to EcoCyc users. The two reactions associated with AzoR (NADH: quinone reductase, FMN dependent / NADH-azoreductase) as represented in EcoCyc are shown below. The top reaction is the physiologically relevant reaction catalysed by AzoR, the bottom reaction is the non-physiological reaction catalysed by the enzyme *in* *vitro*.

The identification of non-physiological DEMs has resulted in improvements to the Pathway Tools software, which now enables reactions to be tagged as non-physiological in order to clarify their role (Figure 3). In addition, the software program for identifying DEMs in EcoCyc was modified to exclude reactions that are tagged as non-physiological ensuring that the compounds involved in these reactions will not be returned by the DEM finder. Compounds of uncertain physiological relevance will continue to be returned by the DEM finder in EcoCyc.

A small number of compounds identified as DEMs were deleted from EcoCyc. In several cases, enzyme-catalysed reactions had been added to EcoCyc based on putative functional assignments for *E. coli* open reading frames reported in the literature [15]. For example, the uncharacterised YnbA protein was annotated in EcoCyc as a diacylglycerol cholinephosphotransferase, catalysing the reaction CDP-choline + 1,2-diacylglycerol <=> CMP + a phosphatidylcholine (E.C 2.7.8.2). This reaction produces phosphatidylcholine, which is a major constituent of eukaryotic membrane phospholipids but is present in only about 10% of all bacterial species and is not present in *E. coli* [16]. The reaction was removed from EcoCyc, but information regarding the initial bioinformatic prediction was retained in the text summary associated with the YnbA protein. Similarly, the annotation of *E. coli acs* as an hydroxycinnamate-CoA ligase (E.C 6.2.1.12) was removed, as no evidence for the presence of the common plant metabolites 4-coumarate and 4-coumaroyl-CoA in *E. coli* could be uncovered.

#### DEMs remaining in the EcoCyc database

The physiologically relevant DEMs remaining in the EcoCyc database are listed in Table 4, and likely represent deficiencies in our knowledge of *E. coli* metabolism. 9 of these compounds lack reactions accounting for their production within the network while a further 17 compounds lack reactions that would account for their consumption. 5 compounds (sulfinoalanine, curcumin, L-rhamnonate, quinate and urate) are possible nutrient sources in *E. coli* K-12. Sulfinoalanine, quinate and urate are included on Biolog phenotype assay plates [17] and growth observations from studies using this system have been collated on the EcoCyc website (see EcoCyc website command Search → Growth Media) while L-rhamnose and curcumin utilization have been documented in the literature. The EcoCyc reactions containing the DEMs and the relevant enzymes (Table 4) have all been experimentally characterised in *E. coli* K-12; literature references are available on the EcoCyc website under the enzyme of interest.

**Table 4:** Dead end metabolites remaining in EcoCyc.

**Dead end metabolite**	**EcoCyc reaction**	**Enzyme**
(E)-3-(methoxycarbonyl)pent-2-enedioate	*trans*-aconitate + *S*-adenosyl-L-methionine → (E)-3-(methoxycarbonyl)pent-2-enedioate + *S*-adenosyl-L-homocysteine	trans-aconitate methyltransferase (*tam*)
2-aminobutyrate	an aminated amine donor + 2-oxobutanoate → 2-aminobutyrate + a deaminated amine donor	valine-pyruvate aminotransferase (*avtA*)
2-aminomalonate-semialdehyde	L-serine + NADP^+^ → 2-aminomalonate-semialdehyde + NADPH + 2H^+^	3-hydroxy acid dehydrogenase (*ydfG*)
2-deoxygluconate; 3-dehydro-2-deoxy-D-gluconate	NAD^+^ + 2-deoxygluconate → NADH + 3-dehydro-2-deoxy-D-gluconate + H^+^	2-deoxy-D-gluconate 3-dehydrogenase (*kduD*)
3-methylcrotonyl-CoA; isovaleryl-CoA	isovaleryl-CoA + an oxidized electron-transfer flavoprotein → 3-methylcrotonyl-CoA + a reduced electron-transfer flavoprotein	isovaleryl-CoA dehydrogenase (*aidB*)
3-sulfinoalanine	3-sulfinoalanine + H_2_O → L-alanine + sulfite + H^+^	L-cysteine desulfurase (*sufS*) / cysteine sulfinate desulfinase (*csdA*)
3-α,12-α-dihydroxy-7-oxo-5-β-cholanate	cholate + NAD^+^ → 3α,12α-dihydroxy-7-oxo-5β-cholan-24-oate + NADH + H^+^	7-α-hydroxysteroid dehydrogenase (*hdhA*)
4-hydroxy-2-oxoglutarate	4-hydroxy-2-oxoglutarate ← glyoxylate + pyruvate	multifunctional 2-keto-3-deoxygluconate 6-phosphate aldolase (*eda*)
aminoacetaldehyde	taurine + 2-oxoglutarate + oxygen → aminoacetaldehyde + sulfite + succinate + CO_2_ + H^+^	taurine dioxygenase (*tauD*)
curcumin	curcumin + NADPH + H^+^ → dihydrocurcumin + NADP^+^	NADPH-dependent curcumin/dihydrocurcumin reductase (*curA*)
tetrahydrocurcumin	dihydrocurcumin + NADPH + H^+^ → tetrahydrocurcumin + NADP^+^	NADPH-dependent curcumin/dihydrocurcumin reductase (*curA*)
diacetyl	(*S*)-2-acetolactate + an oxidized electron acceptor + H^+^ → diacetyl + CO_2_ + a reduced electron acceptor acetoin + NADP^+^ → diacetyl + NADPH + H^+^	*yohF* (predicted oxidoreductase)
ethyl-(2R)-methyl-(3S)-hydroxybutanoate; ethyl-2-methylacetoacetate	ethyl-(2R)-methyl-(3S)-hydroxybutanoate + NADP^+^ → ethyl-2-methylacetoacetate + NADPH + H^+^	β-keto ester reductase (*dkgA*)
GDP-α-D-glucose	GDP-α-D-glucose + H_2_O → β-D-glucose + GDP + H^+^	GDP-mannose mannosyl hydrolase (*gmm*)
hydroxylamine	pyruvate + hydroxylamine → pyruvic oxime + H_2_O hydroxylamine + a reduced electron acceptor → ammonia + an oxidized electron acceptor + H_2_O	hybrid-cluster protein (hcp)
linear dimeric GMP	cyclic di-3',5'-guanylate + H_2_O → linear dimeric GMP + H^+^	cyclic di-GMP phosphodiesterase
L-rhamnonate	L-rhamnonate → 2-keto-3-deoxy-L-rhamnonate + H_2_O	L-rhamnonate dehydratase (*yfaW*)
nicotinamide mononucleotide (reduced) (NMNH)	NADH + H_2_O → NMNH + AMP + 2H^+^	NADH pyrophosphatase (*nudC*)
oxamate	oxamate + carbamoyl-phosphate ← oxalurate + phosphate	-
quinate	NAD(P)^+^ + L-quinate → NAD(P)H + 3-dehydroquinate + H^+^	quinate dehydrogenase (*ydiB*)
S-adenosyl-4-methylthio-2-oxobutanoate	*S*-adenosyl-L-methionine + 7-keto-8-aminopelargonate → S-adenosyl-4-methylthio-2-oxobutanoate + 7,8-diaminopelargonate	7,8-diaminopelargonic acid synthase (*bioA*)
tetrahydromonopterin	tetrahydromonapterin + NADP^+^ ← 7,8-dihydromonapterin + NADPH + H^+^	dihydromonapterin reductase (*folM*)
urate	xanthine + NAD^+^ + H_2_O → urate + NADH + H^+^	xanthine dehydrogenase (*xdhABC*)

For a number of DEMs the literature record provides some hint as to possible solutions for resolution. For example, it has recently been suggested that the dead end metabolite oxamate may be further degraded by a pathway that begins with the action of an oxamate:CoA ligase encoded by the gene *fdrA* [18]. Similarly, it has been suggested that the DEM S-adenosyl-4-methylthio-2-oxobutanoate - a by-product of biotin biosynthesis - could decompose non-enzymatically [19]. Several of the remaining DEMs are already the focus of current research efforts. Linear dimeric GMP is the product of the degradation of the signaling molecule cyclic di-GMP (E.C.3.1.4.52), and five enzymes from *E. coli* K-12 are annotated with this activity. Early research showing that the extremely slow degradation of linear dimeric GMP to GMP in *E. coli* K-12 was unlikely to be relevant *in vivo* [20] has led to the more recent suggestion that linear dimeric GMP may itself have signaling capacity which contributes additional complexity to cellular signaling [21]. Likewise, current research into the *E. coli* AidB protein (annotated in EcoCyc as an isovaleryl-CoA dehydrogenase - E.C.1.3.8.4 – and associated with the DEMs isovaleryl-CoA and methylcrotonyl-CoA) is uncovering a role in the protection from alkylating agents [22].

The DEMs remaining at the completion of this study were also queried within the latest version of the Palsson group’s model of *E. coli* K-12 metabolism – known as iJO1366 [23]. Six metabolites are also DEMs in iJO1366. (E) -3-(methoxycarbonyl) pent-2-enedioate, aminoacetaldehyde, oxamate, S-adenosyl-4-methylthio-2-oxobutanoate and tetrahydromonapterin are metabolites with producing reactions but no consuming reactions (called root no consumption gaps in iJO1366) while sulfinoalanine has a consuming reaction but no producing reaction (root no production gap). The EcoCyc DEMs quinate and urate are not DEMs in iJO1366 which includes orphan reactions (enzymatic activity believed to be present in an organism but without any known coding genes) for uricase activity and quinate transport. The EcoCyc metabolic network has neither of these reactions because we do not yet feel there is compelling evidence that these activities occur in *E. coli* K-12. In iJO1366 the reaction producing 2-aminomalonate-semialdehyde is specified as reversible whereas in EcoCyc the reversibility is unspecified, although the text summary in EcoCyc notes that kinetic analysis [24] suggests the reaction would proceed in the reductive direction (that is, towards the production of 2-aminomalonate-semialdehyde). Finally, neither linear dimeric GMP nor NMNH are present in iJO1366 due to differences in the funtional annotation of *dosP* (a cyclic-di-GMP phosphodiesterase in EcoCyc and a 3’ 5’ cyclic nucleotide phosphodiesterase in iJO1366) and *nudC* (an NADH diphosphatase with NAD^+^ as an alternative substrate in EcoCyc and an NAD^+^ diphosphatase in iJO1366).

## Discussion

The DEM finder tool is able to identify dead end metabolites in the biochemical network of *E. coli* K-12 as represented in the EcoCyc database. Using this program, we identified 127 DEMs within the database, analysed their context and undertook a literature search to see if any could be further resolved. The model organism *E. coli* K-12 is an intensively studied bacterium, much of its biochemical network has been experimentally elucidated, and many of the biochemical pathways represented in EcoCyc are complete – this is reflected in the considerably smaller number of DEMs derived from within pathways (32 compounds from 932 reactions, a rate of .035 DEM per reaction) compared to those derived from isolated reactions (95 compounds from 393 reactions, a rate of .24 DEM per reaction). Our analyses resulted in the direct resolution of the dead-end status of just under half these metabolites.

38 new transport reactions were added to the EcoCyc database. Over half of these could not be assigned to a specific transport protein. In the majority of cases where a transporter was identified, the transport capability reflected an expanded substrate range for a previously characterised protein rather than the assignment of new function to a transporter of unknown substrate specificity. The identification of compounds that are imported into *E. coli* K-12 but are lacking known transport proteins (Table 2) may be of interest to researchers in this field. For example, *E. coli* K-12 can use phenylethylamine as a sole carbon and energy source [25] and a pathway for the degradation of this compound has been characterised (http://ecocyc.org/ECOLI/NEW-IMAGE?type=PATHWAY&object=PWY-6071). A periplasmic amine oxidase (encoded by *tynA*) converts phenylethylamine to phenylacetaldehyde which is the substrate of a cytoplasmic dehydrogenase (encoded by *feaB*) but an inner membrane transport system for phenylacetaldehyde has not been characterised. Transport proteins specific for aromatic compounds such as hydroxyphenylpropionic acid and 3-phenylpropionate and for aromatic amino acids have been characterised in *E. coli* K-12, and perhaps one of these also transports phenylacetaldehyde. The existence of an uncharacterised open reading frame - *ydbH* – predicted to contain beta-barrel structure [26] and located just upstream of the genes encoding proteins involved in phenylethylamine degradation is also noted.

Several compounds with unknown transport systems are substrates of scavenging reactions. *E. coli* can salvage the compounds 4-methyl-5-(β-hydroxyethyl)thiazole (THZ) and hydroxymethylpyrimidine (HMP) for use in thiamine synthesis (http://metacyc.org/META/NEW-IMAGE?type=PATHWAY&object=PWY-6897). A predicted thiamine transporter encoded by the genes *tbpA, thiP* and *thiQ* exists in *E. coli* K-12 but has not been functionally characterised and thus its substrate range is unknown. Similarly, biotin-*d*-sulfoxide can be used as an alternative source of biotin by *E. coli* mutant strains that are unable to synthesize biotin [27]. Active transport of biotin is thought to occur in *E. coli* but no transporter has been identified [28,29].

Our analysis identified reactions within the EcoCyc database that are not physiologically relevant; these reactions and compounds are not part of normal *E. coli* metabolism, however their inclusion in EcoCyc provides useful data regarding enzyme specificity and kinetics. Again, the status of *E. coli* K-12 as a model organism is a contributing factor to the large number of non-physiological compounds that are included in the EcoCyc database. Many of its enzymes have been purified and characterised *in vitro*, sometimes using standard assays that are not relevant *in vivo* or in other cases focusing on enzyme activity and compounds that may be of industrial importance. Our DEM analysis indicated a clear need to better identify these reactions in EcoCyc and to modify the software program that is used to identify DEMs in order to exclude these reactions from future analyses.

DEMs have the potential to affect metabolic modelling, for instance DEM-driven corrections to a metabolic database will improve the accuracy of flux-balance analysis (FBA) models [30] derived from that database (e.g., the flux-balance analysis metabolic model derived from EcoCyc [3]). In FBA models, the fluxes of reactions that produce a metabolite must be balanced by the fluxes of reactions that consume that metabolite. Thus, reactions involving DEMs can never carry flux in FBA models, which decreases model fidelity since under some growth conditions these reactions do carry flux in living cells. For example, the addition of the transport reaction for 1-deoxy-D-xylulose allows the metabolic model to now correctly predict growth of *E. coli* under that substrate, and correction of a DEM in the EcoCyc pathway for adenosylcobalamin (coenzyme B12) salvage will allow that pathway to carry flux.

In theory, DEMs reflect areas where our knowledge of metabolism is lacking and thus may be a useful tool for identifying potential research targets. Achieving this goal relies on accurate identification of physiologically relevant DEMs and our analysis of the output of the DEM finder in EcoCyc has enabled us to improve on the ability to do that for *E. coli* K-12. The improvements and insights we have gained by analysing the DEMs within EcoCyc are likely to prove useful when applied to Pathway/Genome Databases of other organisms. Manual curation of the EcoCyc database is an ongoing process and thus the list of dead end metabolites is not static. As new enzyme functions are characterised and biochemical pathways elucidated, the output of the dead end finder will vary. Evaluation of the DEMs in the database will thus be an ongoing task and an important check on the quality and overall coherence of the information in EcoCyc.

## Methods

The computational definition of DEMs used by the EcoCyc DEM finder is given below. The definition references a specified compartment, including the reactions and transporters present in that compartment, which for the purpose of this study was always the cytoplasm. Substrates in EcoCyc reactions can be specific compounds or they can be compound classes. The term “C” refers to a specific compound while “a parent of C” refers to a compound class to which compound C belongs. For example, the specific compound “cytosine” belongs to the compound class “a pyrimidine base”.

A compound C is a DEM if

1 C or a parent of C is only produced by small-molecule metabolic (SMM) reactions in Compartment, and C or a parent of C is not transported out of Compartment, OR
2 C or a parent of C is only consumed by SMM reactions in Compartment, and C or a parent of C is not transported into Compartment

The DEM finder was also instructed to ignore compounds that are recorded in EcoCyc as enzyme cofactors, since we would expect synthesized cofactors to be used by the cell but not necessarily consumed by metabolic reactions.

Compounds returned by the DEM finder were initially analysed within the context of the EcoCyc database in order to locate the reactions (and associated enzymes) producing or consuming them and to review the literature that led to their inclusion in the database. Comprehensive literature searches were then undertaken to see if further information relating to the metabolism or transport of the compound of interest could be uncovered. Specific searches of *E. coli*-related literature were done using the text mining system, Textpresso [31]. In Textpresso for *E. coli* (see EcoCyc website command Search → Search Full-Text Articles) a user can search, via specific keywords and/or categories, a literature database that currently contains just over 30,000 full text articles and 7000 abstracts. More general searches were also undertaken using publicly available internet search engines.

## Supporting Information

Table S1 **Transport reactions representing the import of a metabolite added to EcoCyc.**
(DOCX)

Table S2 **Transport reactions representing the export of a metabolite added to EcoCyc.**
(DOCX)
