# Supplementary material for: Dead End Metabolites - Defining the Known Unknowns of the E. coli Metabolic Network
Source: PLoS One. 2013 Sep 23;8(9):e75210. doi: 10.1371/journal.pone.0075210 (PMC3781023; doi:10.1371/journal.pone.0075210)
Supplement: Table S1 — Transport reactions representing the import of a metabolite added to EcoCyc. (DOCX) [file pone.0075210.s001.docx]

| **Transport reaction added to EcoCyc** | **Transport system** |
| --- | --- |
| acetoacetate_[periplasmic space]_ → acetoacetate_[cytosol]_ | predicted short chain fatty acid transporter AtoE |
| phenylacetaldehyde_[periplasmic space]_ → phenylacetaldehyde_[cytosol]_ | **-** |
| psicoselysine_[periplasmic space]_ → psicoselysine_[cytosol]_ | psicoselysine transporter FrlA |
| D-fructosyl-L-lysine_[periplasmic space]_ → D-fructosyl-L-lysine_[cytosol]_ | fructoselysine transporter FrlA |
| octanoate_[periplasmic space]_ → octanoate_[cytosol]_ | **-** |
| 3-phenylpropanoate_[periplasmic space]_ → 3-phenylpropanoate_[cytosol]_ | predicted 3-phenylpropanoate transporter HcaT |
| (*R*)-lipoate_[periplasmic space]_ → (*R*)-lipoate_[cytosol]_ | **-** |
| cobinamide_[extracellular space]_ → cobinamide_[cytosol]_ | vitamin B12 transport system |
| 3-hydroxy-*trans*-cinnamate_[periplasmic space]_ + H^+^_[periplasmic space]_ → 3-hydroxy-*trans*-cinnamate_[cytosol]_ + H^+^_[cytosol]_ | 3-hydroxycinnamate:H^+^ symporter MhpT |
| *trans*-cinnamate_[periplasmic space]_ → *trans*-cinnamate_[cytosol]_ | **-** |
| (2R,4S)-2-methyl-2,3,3,4-tetrahydroxytetrahydrofuran_[periplasmic space]_ + ATP + H_2_O → (2R,4S)-2-methyl-2,3,3,4-tetrahydroxytetrahydrofuran_[cytosol]_ + ADP + phosphate + H^+^ | autoinducer-2 ABC transporter LsrACDB |
| 4-methyl-5-(β-hydroxyethyl)thiazole_[periplasmic space]_ → 4-methyl-5-(β-hydroxyethyl)thiazole_[cytosol]_ | **-** |
| 5,6-dimethylbenzimidazole_[periplasmic space]_ → 5,6-dimethylbenzimidazole_[cytosol]_ | **-** |
| hydroxymethylpyrimidine_[periplasmic space]_ → hydroxymethylpyrimidine_[cytosol]_ | **-** |
| pseudouridine_[periplasmic space]_ → pseudouridine_[cytosol]_ | predicted pseudouridine transporter PsuT |
| ethanolamine_[periplasmic space]_ → ethanolamine_[cytosol]_ | **-** |
| allantoin_[periplasmic space]_ → allantoin_[cytosol]_ | predicted transporter YbbW |
| (R)-malate_[periplasmic space]_ + 2 H^+^_[periplasmic space]_ → (R)-malate_[cytosol]_ + 2 H^+^_[cytosol]_ | C4 dicarboxylate/orotate transporter DctA |
| 1-deoxy-D-xylulose_[periplasmic space]_ → 1-deoxy-D-xylulose_[cytosol]_ | **-** |
| *d*-biotin *d*-sulfoxide_[periplasmic space]_ → *d*-biotin *d*-sulfoxide_[cytosol]_ | **-** |
| glycerol 2-phosphate_[periplasmic space]_ + ATP + H_2_O → glycerol 2-phosphate_[cytosol]_ + ADP + phosphate + H^+^ | glycerol-3-phosphate/glycerol-2-phosphate ABC transporter |
| 1-(β-D ribofuranosyl)nicotinamide_[periplasmic space]_ → 1-(β-D ribofuranosyl)nicotinamide_[cytosol]_ | nicotinamide riboside ABC transporter |
| S-methyl-L-methionine_[periplasmic space]_ → S-methyl-L-methionine_[cytosol]_ | S-methyl-L-methionine transporter |
| selenite_[periplasmic space]_ + ATP + H_2_O → selenite_[cytosol]_ + ADP + phosphate + H^+^ | sulfate/thiosulfate/selenite/selenate ABC transporter |
| selenate_[periplasmic space]_ + ATP + H_2_O → selenate_[cytosol]_ + ADP + phosphate + H^+^ | sulfate/thiosulfate/selenite/selenate ABC transporter |
| L-selenocysteine_[periplasmic space]_ → L-selenocysteine_[cytosol]_ | **-** |
| cholate_[periplasmic space]_ → cholate_[cytosol]_ | **-** |
| L-glyceraldehyde 3-phosphate_[periplasmic space]_ + phosphate_[cytosol]_ → L-glyceraldehyde 3-phosphate_[cytosol]_ + phosphate_[periplasmic space]_; L-glyceraldehyde 3-phosphate_[periplasmic space]_ → L-glyceraldehyde 3-phosphate_[cytosol]_ | glycerol 3-phosphate transport systems |
| an aminoalkylphosphonate_[periplasmic space]_ + ATP + H_2_O → an aminoalkylphosphonate_[cytosol]_ + ADP + phosphate + H^+^ | phosphonate ABC transporter |

**Table S1**: Transport reactions representing the import of a metabolite added to EcoCyc
