# Supplementary material for: Dead End Metabolites - Defining the Known Unknowns of the E. coli Metabolic Network
Source: PLoS One. 2013 Sep 23;8(9):e75210. doi: 10.1371/journal.pone.0075210 (PMC3781023; doi:10.1371/journal.pone.0075210)
Supplement: Table S2 — Transport reactions representing the export of a metabolite added to EcoCyc. (DOCX) [file pone.0075210.s002.docx]

| **Transport reaction added to EcoCyc** | **Transport system** |
| --- | --- |
| (3E,5Z)-tetradecadienoate_[cytosol]_ → (3E,5Z)-tetradecadienoate_[periplasmic space]_ | **-** |
| 3-hydroxypropionate_[cytosol]_ → 3-hydroxypropionate_[extracellular space]_ | **-** |
| *S*-methyl-5-thio-D-ribose_[cytosol]_ → *S*-methyl-5-thio-D-ribose_[periplasmic space]_ | **-** |
| urea_[cytosol]_ ↔ urea_[periplasmic space]_ | glycerol channel GlpF; passive diffusion |
| methanol_[cytosol]_ → methanol_[periplasmic space]_ | **-** |
| (2R,4S)-2-methyl-2,3,3,4-tetrahydroxytetrahydrofuran_[cytosol]_ → (2R,4S)-2-methyl-2,3,3,4-tetrahydroxytetrahydrofuran_[periplasmic space]_ | quorum signal AI-2 exporter TqsA |
| acetylmaltose_[cytosol]_ → acetylmaltose_[extracellular space]_ | **-** |
| salicyl alcohol_[cytosol]_ → salicyl alcohol_[periplasmic space]_ | **-** |
| benzene-1,4-diol_[cytosol]_ → benzene-1,4-diol_[periplasmic space]_ | **-** |

**Table S2**: Transport reactions representing the export of a metabolite added to EcoCyc
